# sEVD—smartphone-navigated placement of external ventricular drains

**DOI:** 10.1007/s00701-019-04131-9

**Published:** 2019-11-25

**Authors:** Christian V. Eisenring, Felice Burn, Michelle Baumann, Lennart H. Stieglitz, Ralf A. Kockro, Jürgen Beck, Andreas Raabe, Markus F. Oertel

**Affiliations:** 1 Department of Neurosurgery, Inselspital, Bern University Hospital, University of Bern, Freiburgstrasse 10, 3010 Bern, Switzerland; 2 Department of Neurosurgery, University Medicine Goettingen, Georg-August-University Goettingen, Goettingen, Germany; 3 Department of Neurosurgery, Hirslanden Hospital, Zurich, Switzerland; 4 Institute of Radiology and Neuroradiology, Cantonal Hospital Aarau, Aarau, Switzerland; 5 Department of Neurosurgery, University Hospital Zurich, University of Zurich, Zurich, Switzerland; 6 Clinical Neuroscience Center, University Hospital Zurich, University of Zurich, Zurich, Switzerland; 7 Department of Neurosurgery, University Medical Center Freiburg, University of Freiburg, Freiburg, Germany

**Keywords:** App, External ventricular drain, Neuronavigation, Smartphone, Ventriculostomy

## Abstract

**Background:**

Currently, the trajectory for insertion of an external ventricular drain (EVD) is mainly determined using anatomical landmarks. However, non-assisted implantations frequently require multiple attempts and are associated with EVD malpositioning and complications. The authors evaluated the feasibility and accuracy of a novel smartphone-guided, angle-adjusted technique for assisted implantations of an EVD (sEVD) in both a human artificial head model and a cadaveric head.

**Methods:**

After computed tomography (CT), optimal insertion angles and lengths of intracranial trajectories of the EVDs were determined. A smartphone was calibrated to the mid-cranial sagittal line. Twenty EVDs were placed using both the premeasured data and smartphone-adjusted insertion angles, targeting the center of the ipsilateral ventricular frontal horn. The EVD positions were verified with post-interventional CT.

**Results:**

All 20 sEVDs (head model, 8/20; cadaveric head, 12/20) showed accurate placement in the ipsilateral ventricle. The sEVD tip locations showed a mean target deviation of 1.73° corresponding to 12 mm in the plastic head model, and 3.45° corresponding to 33 mm in the cadaveric head. The mean duration of preoperative measurements on CT data was 3 min, whereas sterile packing, smartphone calibration, drilling, and implantation required 9 min on average.

**Conclusions:**

By implementation of an innovative navigation technique, a conventional smartphone was used as a protractor for the insertion of EVDs. Our ex vivo data suggest that smartphone-guided EVD placement offers a precise, rapidly applicable, and patient-individualized freehand technique based on a standard procedure with a simple, cheap, and widely available multifunctional device.

## Introduction

Insertion of an external ventricular drain (EVD) is one of the most commonly performed procedures in daily neurosurgical practice [6, 7]. The standard technique is still the traditional non-navigation-assisted freehand placement exclusively guided by anatomical landmarks [2, 7, 28]. By this method, an EVD is usually inserted at Kocher’s point [9] and directed towards the nasion in the sagittal plane, and the tragus or slightly anterior in the coronal plane [13, 23]. However, imprecise EVD placement often occurs [3].

The desire for greater precision of ventriculostomy has led to various technical innovations and use of additional gadgets such as neurosonography [24], frameless stereotaxy [27], endoscopy [29], guiding protractors [8, 12], robotic [16] or electromagnetic neuronavigation [17], and fluoroscopy [4] or CT guidance [7]. Even a smartphone augmented-reality mobile device application (app), with or without a ventricular catheter-guiding tool, has already been developed [5, 25, 28].

Although all these techniques may lead to improved insertion accuracy, they are also associated with various disadvantages including additional time to perform the procedures, the need for head fixation, higher costs of materials, lack of ubiquitous availability of appropriate equipment, as well as time and effort for training and handling the systems. Moreover, neurosurgeons prefer methods that are quick and easy to use and barely differ from established clinical routine [24].

Taking these considerations into account and encouraged by results from an iPod-based navigation procedure described previously [11, 18], we developed a novel smartphone-guided, angle-adjusted technique for implantation of an EVD (sEVD). We systematically assessed its feasibility and accuracy in both a human artificial head model and a cadaver head.

## Methods and materials

### CT-based trajectory planning

A total of 20 EVD trajectories in a human artificial gel-filled head model with inserted ventricles (Classic Human Skull Model; 3B Scientific GmbH, Hamburg, Germany) (8/20) and a human formalin-fixed cadaveric head (Institute of Anatomy, University of Bern, Bern, Switzerland) (12/20) were determined on native multiplanar reformatted CT images (Somatom Definition Edge; Siemens Healthcare, Forchheim, Germany). A commercially available picture archiving and communication system (PACS) workstation (IDS7, Sectra AB, Linköping, Sweden) was used to analyze the images. In the PACS workstation, the native images of the cadaveric head and the head model were displayed in 3 planes: sagittal, coronal, and transversal. Each of 8 different insertion point was measured as determined on the head model 2.5 cm lateral to the midline and on a trajectory towards the upper edge of the external acoustic meatus at 10, 11, 12, and 13 cm posterior to the nasion. On the cadaveric head, 12 insertion points were measured as determined 2 and 3.5 cm paramedially, and 10, 12, and 14 cm behind the nasion, respectively. For each determined insertion point, the coronal image plain was adjusted to include three external landmarks: the insertion point as well as the upper edge of the tragi bilaterally. These three points represented the coronal insertion plain. There, the center of the ipsilateral frontal horn according to the sagittal and transversal ventricle diameters represented the optimal target for the sEVD tip. A trajectory was drawn from the insertion point on the skin of the human’s head and surface of the head model, respectively, to the center of the ipsilateral ventricle.

**Fig. 1:**
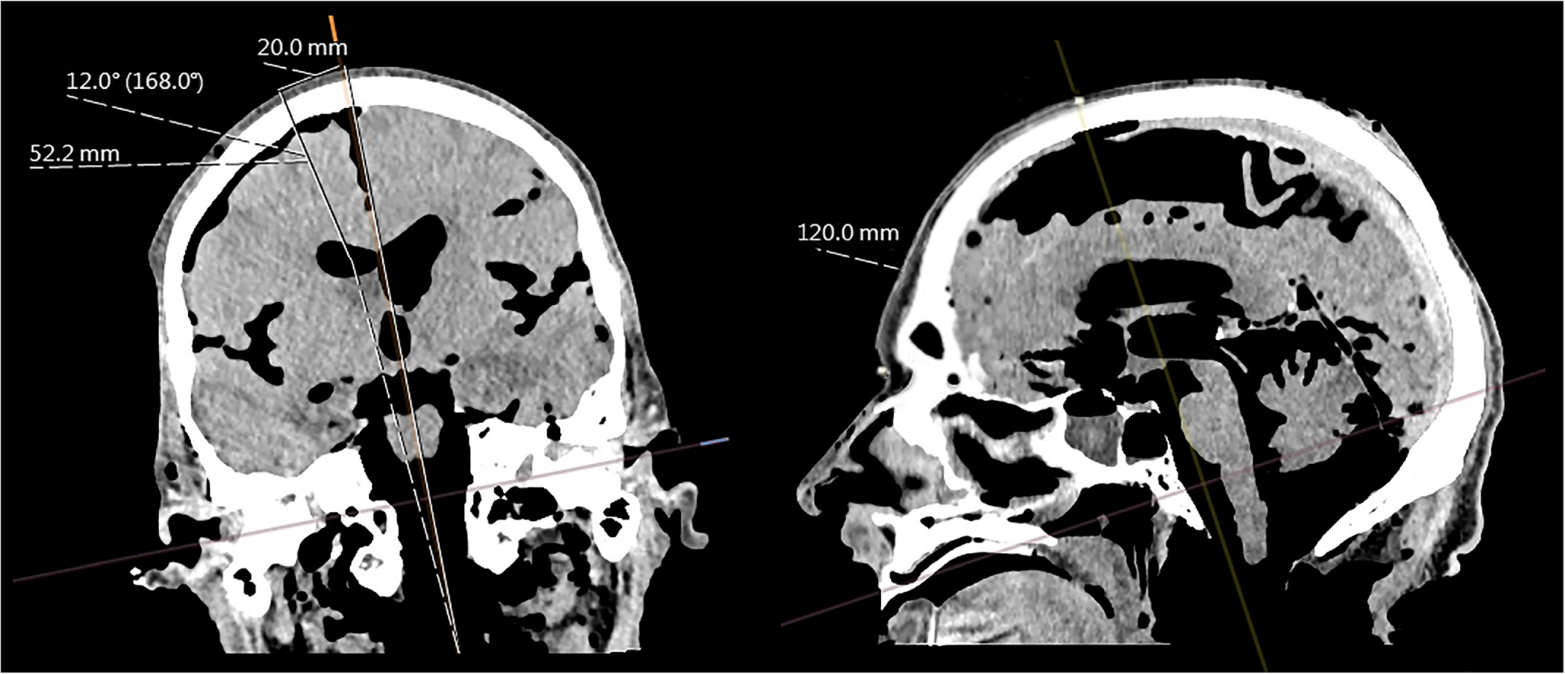
**Planning of sEVD insertion trajectories on multiplanar 3D reformatted native CT.** Coronal (left) and sagittal (right) images of a human cadaveric head demonstrate determination of the sEVD insertion point located 12 cm distally to the nasion and 2 cm paramedially. The trajectory shown is tilted 12° laterally with a 5.22-cm insertion length. Note post-mortem intracranial air inclusion after anatomical fixation and loss of cerebrospinal fluid

Finally, the angles between the sagittal midline and the trajectory, and the distances between the cranial entry point and the center of the ventricle, were measured as depicted in Fig. 1. The angle and the distance between entry point and target point in the center of the ipsilateral ventricle were noted to be at hand for the EVD placement.

### Preparations for EVD placement

A smartphone (iPhone 4S, iOS 7.1.2; Apple Inc., Cupertino, CA, USA) was encased in a sterile cover (SteriPhone; Merete Medical GmbH, Berlin, Germany). A commercially available angle-measuring app (Clinometer; Plaincode App Development, Stephanskirchen, Germany) was installed on the smartphone as a protractor tool.

The heads (model and cadaver) were placed supine. The mid-cranial sagittal line and the insertion points were marked on the surface of both the model and the head. With a measuring tape, the insertion points determined were marked on the surface of both the model and cadaveric head. Additionally, electrocardiographic adhesive patches were placed on the nasion, the midline, and the tragus serving as palpable fiducials. To simulate clinical conditions, drapes were taped around Kocher’s point. The anatomical reference points were finally marked and a line between each insertion point and the upper edge of the external acoustic meatus was drawn on the drapes. This curvilinear line represented the shortest distance from the insertion point to the tragus. This line was adjusted at the CT-based trajectory planning in the sagittal and coronal plane as described above (Fig. 1).

For sEVD placement, the surgeon’s viewing direction was orientated on the coronal insertion plane including the curvilinear line, thereby the curvilinear line appeared straight. The base of the smartphone was positioned on this line and the surface of the smartphone-display was aligned to the coronal insertion plane allowing the most accurate sEVD insertion.

### EVD placement

**Fig. 2:**
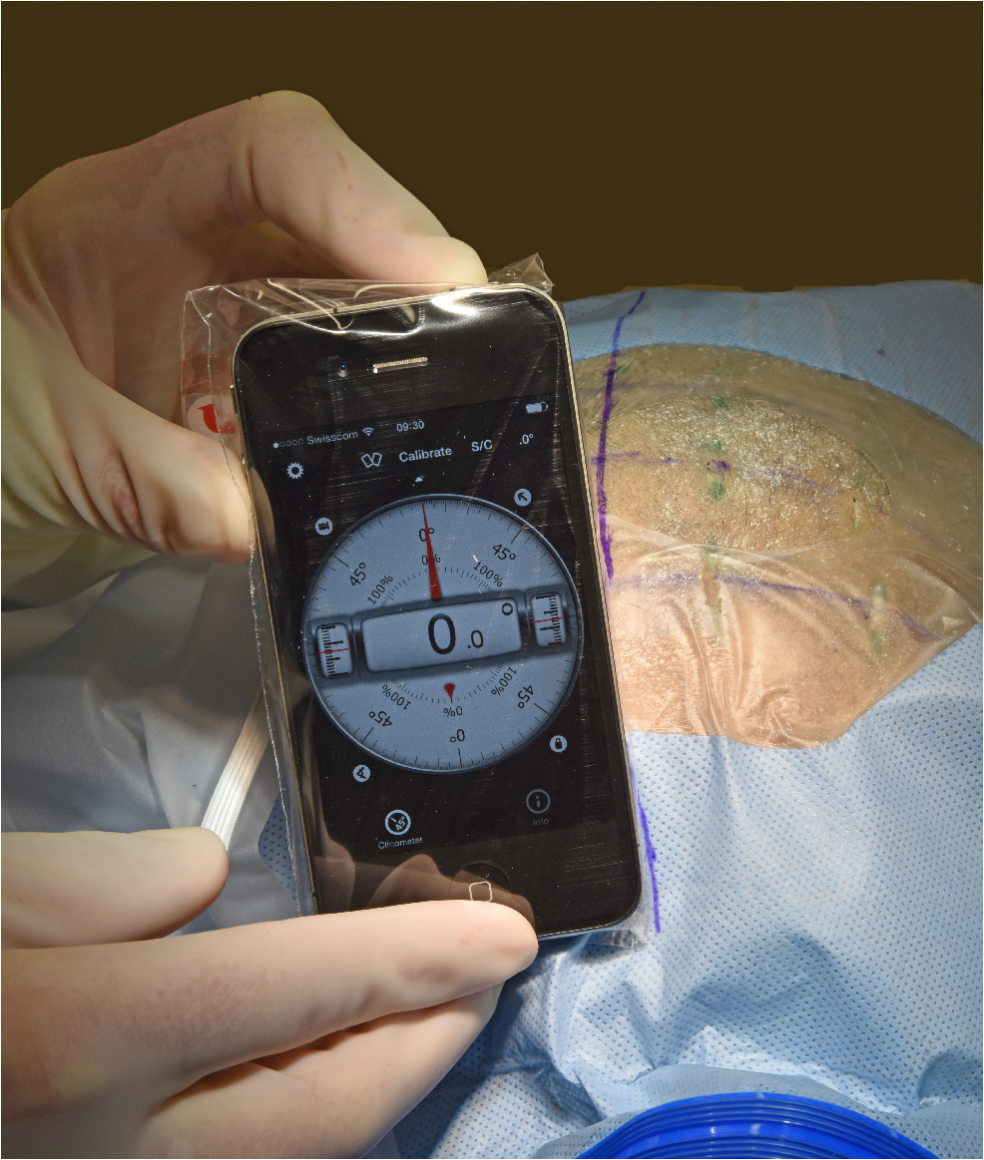
Smartphone calibration. The 0° angle was calibrated according to the sagittal midline. The smartphone was placed along the midline parallel to the insertion plane defined by both the upper edge of the external acoustic meatus and Kocher’s point

The longitudinal smartphone axis was aligned with the marked midline in the coronal insertion plane calibrated as 0° lateral deviation with the app (Fig. 2). The smartphone could be set to announce the lateral angle verbally, obviating visual control of the display. Especially because the angle-measuring app is still investigational and not labeled for neurosurgical use thus far, the accurate angle specification of the app was additionally verified by tilting the iPhone along the set-up triangle with specified angles.

Moreover, on the cadaveric head, a linear incision of 1 cm was made at each insertion point. With a twist drill (Cranial Access Kit; Integra NeuroSciences, Plainsboro, NJ, USA) a burr hole trephination was carried out.

**Fig. 3:**
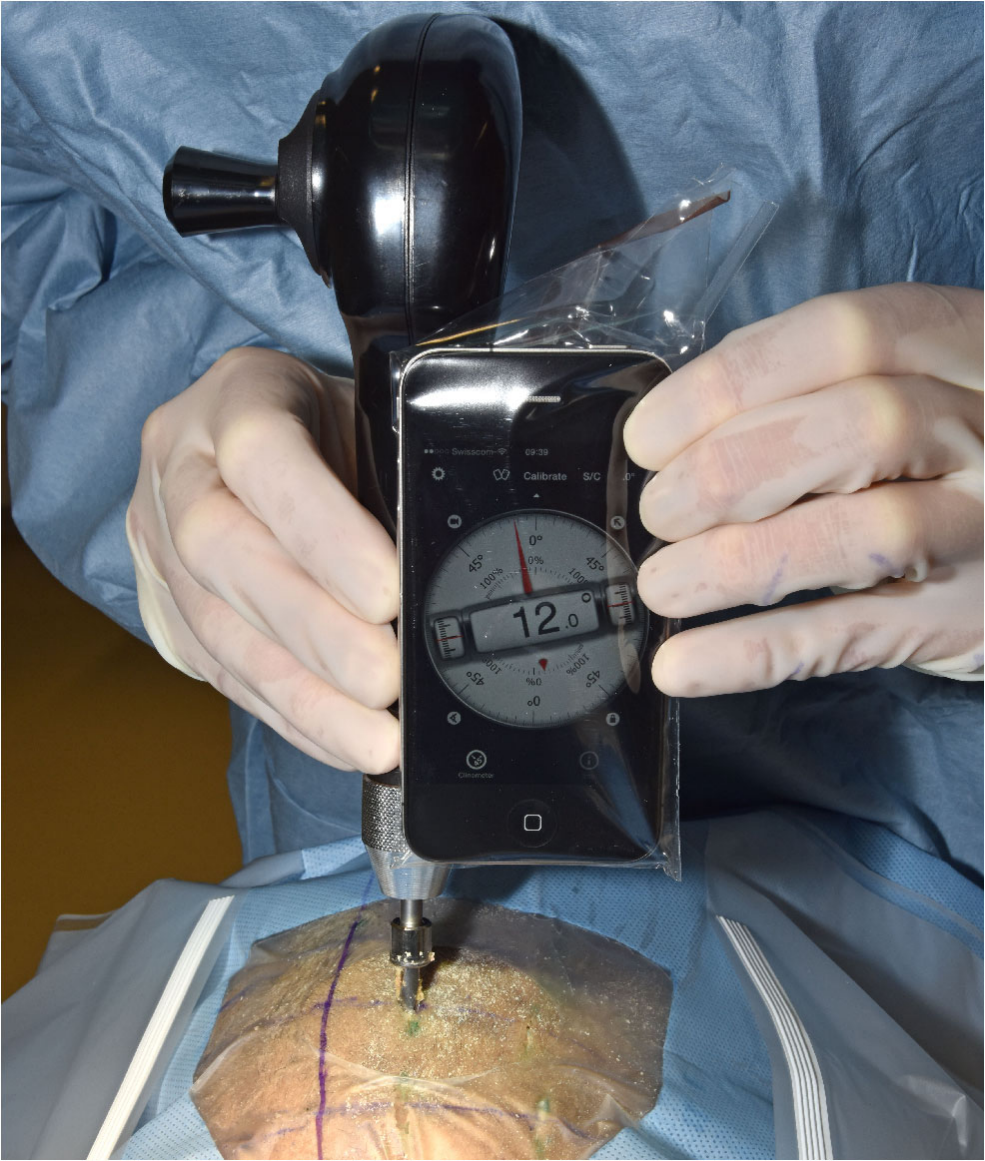
Marking of midline in the sagittal plane, determination of the coronal insertion plan and angle-adjusted trephination. The insertion plane is represented by the curvilinear line that crosses the midline and the insertion point. The axis of the drill was routinely guided within the coronal insertion plane under visual control. It was tilted laterally toward the planned angle with the aid of the smartphone apps verbal angle specification. Placement of the twist drill was followed by angle-adjusted trephination

One surgeons hand held the smartphone whereas the other hand attached the twist drill to it. The angulation of the drill was measured and adjusted with the protractor app of the smartphone orientated along the predefined coronal insertion plane (Fig. 3). Thus, the smartphone could be tilted laterally, but still remained in the same insertion plane, and the lateral angle and the twist drill could be controlled and adjusted.

After trephination, the dura of the cadaveric head was perforated. The EVD (Bactiseal EVD Catheter system; Codman, Raynham, MA, USA) was tightly attached to the longitudinal axis of the smartphone and the planned lateral insertion angle was adjusted.

**Fig. 4:**
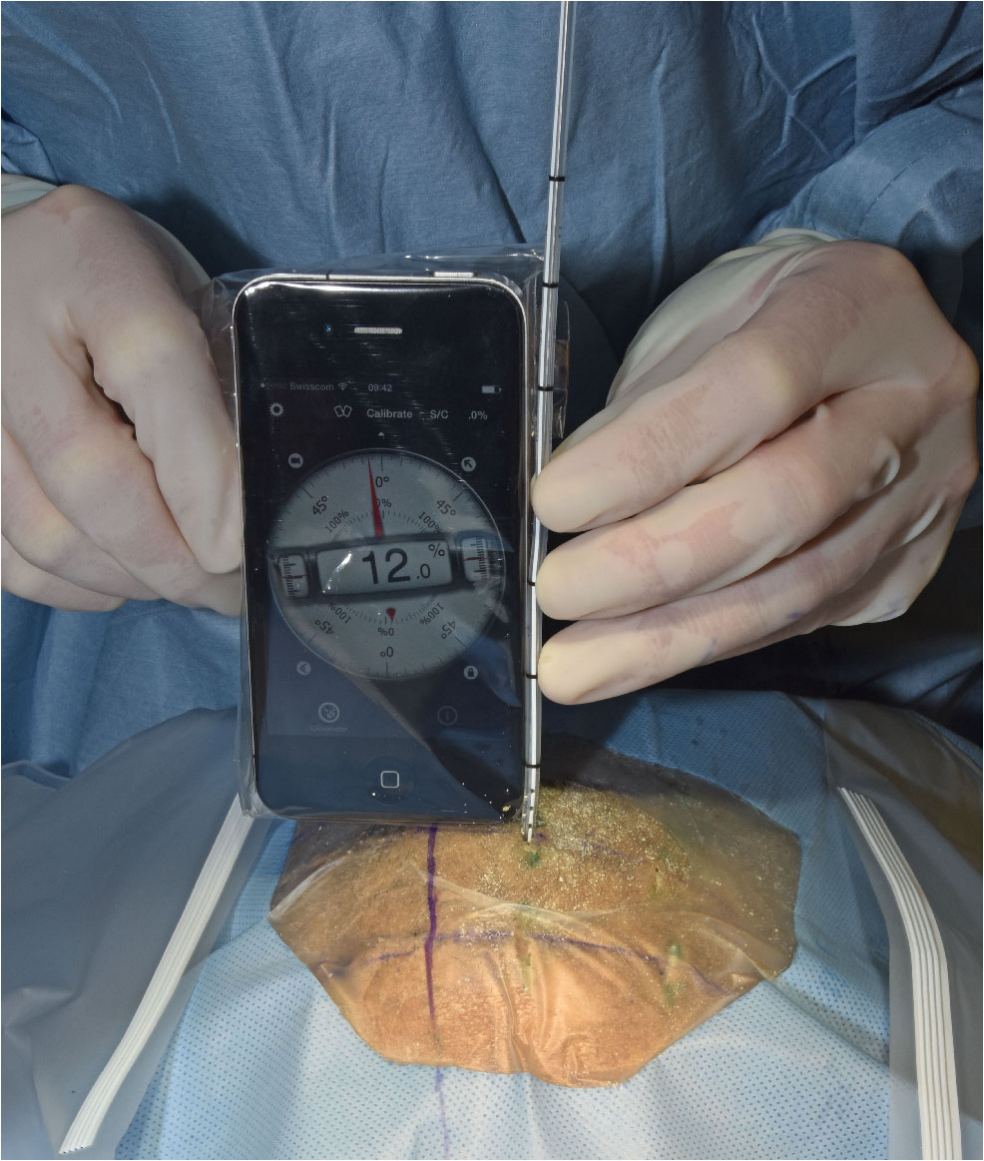
Smartphone-navigated placement of the EVD. The drain was angle-adjusted with the smartphone and inserted into the ventricle. The midline is marked in the sagittal plane. The insertion plane is represented by the curvilinear line that crosses the midline and the insertion point. The axis of the catheter was always guided within the coronal insertion plane under visual control. It was tilted laterally toward the planned angle with the aid of the smartphone apps verbal angle specification

Each sEVD was inserted along the predetermined distance of the scaled catheter (Fig. 4). Durations of all procedures from the time of skin incision to final sEVD insertion in the head were measured.

### Post-interventional assessment

To analyze the results in the model and head, pre- and post-insertion CT images were used to assess accuracy and precision of sEVD insertions in a digital imaging and communications in medicine (DICOM) viewer. First, the images were multiplanar 3D reformatted. Then, the final position of each catheter insertion point, the planned trajectory, and the intracranial positions of the tips were marked. The insertion angle towards the planned trajectory and the lateral deviation in the coronal plane were determined. In addition, the deviation of the catheter tip from the target trajectory in the center of the ventricle was measured. The position of the catheter tips was graded as located either ipsi- or contra-lateral, and intra- or extra-ventricular. Occurrence of kinking was also noted. Additionally, the accuracy of each burr hole location behind the nasion and lateral to the midline and the actual intracranial insertion depth of the catheters was measured.

### Data analysis

Results are provided as both mean values and standard deviation (SD). The following parameters were calculated: planned pre-insertional lateral implantation angles, inserted catheter lengths, locations of catheter tips and burr holes, and their accuracy and deviations from the optimal trajectories measured on CT. Statistical analyses were performed using the commercially available software SPSS Statistics, Version 22 (IBM Corp, Armonk, NY, USA).

## Results

### Head model

In the artificial head, the optimal lateral pre-insertional angles planned in the coronal plane averaged 10.54° (SD ± 5.37, range 4° to 19.6°), whereas the average angles measured on post-interventional CT imaging were 11.34° (SD ± 4.34, range 0.69° to 19.4°). After insertion, the mean deviation from the pre-insertional planned lateral angles was 1.73° (SD ± 1.12, range 0.15° to 3.1°). The post-interventional CT imaging showed that the catheter tips deviated on average 0.12 cm (SD ± 0.08, range 0.01–0.22 cm) from the initially planned optimal trajectory (Table 1).

**Table 1:** Planned pre- and post-interventional catheter positions with mean values and standard deviation

	Planned angle (degrees)	Post-interventional angle (degrees)	Deviation (degrees)	Deviation (cm)	Intraventricular position	Kinking
Head model						
	19.60	19.40	0.20	0.01	+	−
	16.95	16.80	0.15	0.01	+	−
	14.05	13.10	0.95	0.07	+	−
	10.35	7.90	2.45	0.16	+	−
	7.70	10.20	2.50	0.17	+	−
	5.35	8.50	3.15	0.22	+	−
	6.35	7.90	1.55	0.11	+	−
	4.00	6.90	2.90	0.21	+	−
*M*	*10.54*	*11.34*	*1.73*	*0.12*		
*SD*	*5.37*	*4.34*	*1.12*	*0.08*		
Cadaveric head						
	18.70	16.50	2.20	0.21	+	−
	49.30	42.10	7.20	0.81	+	+
	13.00	13.90	0.90	0.09	+	−
	43.00	45.80	2.80	0.25	+	−
	10.00	9.30	0.70	0.06	+	−
	40.40	37.20	3.20	0.26	+	+
	12.00	18.10	6.10	0.60	+	+
	42.00	45.90	3.90	0.35	+	+
	13.20	10.80	2.40	0.21	+	−
	37.00	30.70	6.30	0.54	+	+
	8.00	12.00	4.00	0.39	+	−
	35.00	36.70	1.70	0.15	+	−
*M*	*26.8*	*26.58*	*3.45*	*0.33*		
*SD*	*14.87*	*13.88*	*2.04*	*0.21*		

*cm* centimeter, *M* mean, *SD* standard deviation

All the sEVD burr holes were located on average 0.16 cm (SD ± 0.1, range 0–0.24 cm) more anteriorly and 0.04 cm (SD ± 0.02, range 0.01–0.07 cm) more medially than planned pre-interventionally. The planned mean optimal trajectory length was 4.1 cm (SD ± 0.12, range 3.9–4.3 cm) and, on post-insertional CT imaging, it averaged 4.03 cm (SD ± 0.13, range 3.8–4.23 cm). The mean deviation of the inserted catheter lengths was 0.13 cm (SD ± 0.12, range 0.04–0.4 cm) (Table 2). No instances of catheter kinking were observed after sEVD placement in the head model.

**Table 2:** Pre-and post-interventional measurements of insertion points posterior to nasion, lateral to midline and insertion depth of the catheter, with mean values and standard deviation for both the head model and human head

	Pre-interventional measurements posterior to nasion (cm)	Deviation of post-interventional measurements posterior to nasion (cm)	Pre-interventional planned position lateral to midline (cm)	Deviation of post-interventional measurements lateral to midline (cm)	Pre-interventional planned insertion depth (cm)	Post-interventional measurement of insertion depth (cm)	Deviation of post-interventional measured insertion depth (cm)
Head model							
	10.00	0.00	2.50	0.06	3.90	3.94	0.04
	11.00	0.20	2.50	0.05	4.30	4.03	0.27
	12.00	0.20	2.50	0.03	4.20	4.14	0.06
	13.00	0.24	2.50	0.04	4.20	3.80	0.40
	10.00	0.00	2.50	0.02	4.00	3.92	0.08
	11.00	0.20	2.50	0.03	4.03	3.99	0.04
	12.00	0.20	2.50	0.01	4.10	4.15	0.05
	13.00	0.24	2.50	0.07	4.10	4.23	0.13
*M*	*11.50*	*0.16*	*2.50*	*0.04*	*4.10*	*4.03*	*0.13*
*SD*	*1.20*	*0.09*	*0*	*0.02*	*0.12*	*0.13*	*0.12*
Cadaveric head							
	10.00	0.60	2.00	0.10	6.05	5.60	0.45
	10.00	0.60	2.00	0.06	6.45	6.64	0.19
	10.00	0.60	5.00	0.19	5.50	5.59	0.09
	10.00	0.60	5.00	0.08	5.01	5.55	0.54
	12.00	0.70	2.00	0.03	5.14	5.30	0.17
	12.00	0.70	2.00	0.07	4.70	5.45	0.75
	12.00	0.70	5.00	0.12	5.22	5.98	0.76
	12.00	0.70	5.00	0.05	5.15	5.59	0.44
	14.00	0.81	2.00	0.01	5.15	5.00	0.15
	14.00	0.81	2.00	0.04	4.90	5.10	0.20
	14.00	0.81	5.00	0.06	5.60	6.20	0.60
	14.00	0.81	5.00	0.03	5.15	5.59	0.44
*M*	*12.00*	*0.70*	*3.50*	*0.07*	*5.34*	*5.63*	*0.40*
*SD*	*1.71*	*0.09*	*1.50*	*0.05*	*0.50*	*0.44*	*0.23*

*cm* centimeter, *M* mean, *SD* standard deviation

### Cadaveric head

In the human head, the optimal lateral pre-insertional angles planned in the coronal plane averaged 26.8° (SD ± 14.87, range 8° to 49.3°), whereas the post-interventional angles measured on CT imaging were 26.58° (SD ± 13.88, range 9.3° to 45.9°). After insertion, the mean deviation from the planned pre-insertional lateral angles was 3.45° (SD ± 2.04, range 0.7° to 7.2°) (Table 1). The post-interventional CT imaging showed that the catheter tips deviated on average 0.33 cm (SD ± 0.21, range 0.06–0.81 cm) from the initially planned optimal trajectory (Table 1).

All the sEVD burr holes were located on average 0.7 cm (SD ± 0.09, range 0.6–0.81 cm) more anteriorly and 0.07 cm (SD ± 0.05, range 0.01–0.19) more medially than planned pre-interventionally. The planned mean optimal trajectory length was 5.34 cm (SD ± 0.5, range 4.7–6.45 cm) and, on post-insertional CT imaging, it averaged 5.63 cm (SD ± 0.44, range 5.0–6.64 cm). The mean deviation of the catheter lengths was 0.4 cm (SD ± 0.23, range 0.09–0.76 cm) (Table 2).

On post-interventional CT imaging, we observed 5 sEVDs with intracranial kinking (Table 1). These cases were excluded from further analysis. In the remaining 7 cases, the planned optimal lateral pre-insertional angles in the coronal plane averaged 20.13° (SD ± 12.51, range 8–43°), whereas post-interventional angles measured on CT imaging were 20.70° (SD ± 13.38, range 9.3–45.8°). After insertion, the mean deviation from the planned pre-insertional lateral angles was 2.1° (SD ± 1.05, range 0.7–4°). The catheter tips deviated on average 0.19 cm (SD ± 0.10, range 0.06–0.39 cm) from the optimal trajectory planned (Table 3). The planned mean optimal trajectory lengths were on average 5.37 cm (SD ± 0.34, range 5.01–6.05 cm) and on post-insertional imaging controls averaged 5.54 cm (SD ± 0.34, range 5.0–6.2 cm). The mean deviation of the catheter lengths was 0.35 cm (SD ± 0.19, range 0.09–0.6 cm) (Table 3).

**Table 3:** Planned pre- and post-interventional catheter positions after exclusion of catheters with kinking, with mean values and standard deviation in the cadaveric head

	Planned angle (degrees)	Post-interventional angle (degrees)	Deviation (degrees)	Deviation (cm)	Pre-interventional planned insertion depth (cm)	Post-interventional measurement of insertion depth (cm)	Deviation of post-interventional measured insertion depth (cm)
	18.70	16.50	2.20	0.21	6.05	5.60	0.45
	13.00	13.90	0.90	0.09	5.50	5.59	0.09
	43.00	45.80	2.80	0.24	5.01	5.55	0.54
	10.00	9.30	0.70	0.06	5.13	5.30	0.17
	13.20	10.80	2.40	0.21	5.15	5.00	0.15
	8.00	12.00	4.00	0.39	5.60	6.20	0.60
	35.00	36.70	1.70	0.15	5.15	5.59	0.44
*M*	*20.13*	*20.70*	*2.10*	*0.19*	*5.37*	*5.54*	*0.35*
*SD*	*12.51*	*13.38*	*1.05*	*0.10*	*0.34*	*0.34*	*0.19*

*cm* centimeter, *M* mean, *SD* standard deviation

### Duration of procedure

The mean time taken for preoperative radiological measurement of planned cranial insertion points, lateral angles in the coronal plane, and optimal trajectory lengths was 3.23 min (SD ± 0.57, range 2.8–5 min) in both the human head model and the cadaveric head.

Only the human cadaveric head was used for determination of drilling time due to its comparability with real clinical conditions. The total time required for skin incisions, bone drilling, dura perforation, and smartphone-assisted insertion of the sEVDs was 108 min for 12 sEVDs, and 9 min on average for each insertion (SD ± 1.11, range 8.3–12 min).

## Discussion

In daily neurosurgical practice, especially in emergency settings with critically ill patients, the patient’s outcome might substantially depend on immediate, safe, and sufficient EVD implantations [10]. Therefore, an accurate and rapid procedure is essential to reduce the consequences of uncontrolled intracranial pressure and associated morbidity or mortality [7, 14].

### Ventricular diameter, angle, and target deviation and duration of procedure

An average anatomical diameter of unilateral ventricular frontal horns between 2.61 and 3.78 cm was determined in 150 healthy subjects [22]. In subgroups with pathological narrowing, the unilateral ventricular diameters may range from only 0.1 to 1.6 cm [16]. In contrast, in hydrocephalic patients, the bifrontal distances could be longer, varying from 2.6 to 4.8 cm [12].

In the present study, we measured a mean sEVD tip deviation from the target point of 0.33 cm in the human cadaveric head, including cases with kinking (12/20). After exclusion of catheters with kinking, the deviation of the remaining 7/20 sEVDs target was even less (0.19 cm).

Earlier studies on conventional, non-assisted freehand techniques have reported a mean deviation from the target of 0.97 cm [19], 1.43 cm [7], and 1.6 cm [10]. In studies on freehand ventriculostomy, a lateral angular variation of up to 5° in 51% and up to 15° in 90% of cases was documented by Abdoh et al. [1] Using CT-guided insertion, Gautschi et al. reported a deviation from the target of 0.96 cm [7]. O’Leary and colleagues documented 0.37-cm deviation with the Ghajar-Guide [19], whereas Reinertsen et al. reported a deviation from the target of less than 0.3 cm with a 3D-ultrasound-guidance for EVD insertions [24]. An average deviation of 0.16 cm of the EVD tip from the planned target in a study of smartphone-supported navigation planning, and use of a ventricular catheter-guiding tool was reported by Thomale and co-workers [28]. Finally, both Lollis et al. and Stieglitz and co-workers reported a mean distance of the catheter tip from the target of 0.15 cm after frameless navigated catheter insertion [16, 27]. Regarding the assignment of the determined entry point between CT and head, the plausibility of surface-matching using anatomical landmarks was expressed with a mean error of 0.35 to 0.5 cm [20, 21, 30]. The inaccuracy of angle measurement was correspondingly lower.

### Duration of procedure

In the present study, measurements of the optimal insertion angle and intracranial length of catheters lasted on average 3.23 min. Gautschi et al. reported an average time of 3.8 min for planning of a neuronavigation-assisted EVD placement in a cadaver study [7].

Our average EVD insertion time-including drilling, durotomy, and smartphone-assisted insertion-was 9 min. Mahan et al. reported a total of 17 min on average, whereas Krötz and his colleagues reported a duration of 17 to 20 min for conventional EVD insertion [15, 17]. Furthermore, optimal placement of the catheter in a single pass compensates for the time for trajectory planning and decreases procedure-related complications [7, 14, 26, 31].

### Advantages

Young neurosurgeons especially junior residents with limited experience very often perform insertions of an EVD while a successful implantation preferably at first attempt is imperative to reduce patient’s morbidity. An adequate preparation can essentially help to achieve this aim. The main advantage of the simple method described here is that it increases the probability of accurate one-time EVD insertion, especially if the ventricles are narrow, dislocated, or deformed. Furthermore, the technique applied is easy to learn, not time-consuming, corresponds well to previous conventional EVD implantation methods, and requires barely additional equipment and costs. Basically, the EVD may be inserted as usually, but the aligned and sterile-packed smartphone provides the possibility to display the optimal lateral insertion angle and this increases accuracy and patient safety.

## Limitations

There are several important study limitations and caveats. First, the technique described has been evaluated exclusively in an ex vivo artificial human head model and formalin-fixed human cadaveric head. Although, in principle, we would expect our method to be transferable to clinical application, because smartphone-guided angle-control merely adds a sterile smartphone to conventional freehand insertions, its accuracy would need to be tested in a real clinical setting. Second, although our preliminary results are promising and suggest that the proposed method can offer a supplementary or alternative option to freehand ventricular catheterization, our values might be subject to bias. Both artificial head models and formalin-fixed human heads have a higher consistency than in a living human, contain no cerebrospinal fluid, but rather intraventricular air, increased atrophies, indurated sulci, and carry a higher risk of catheter kinking. The problem of kinking has already been reported as being associated with a significantly increased target deviation risk when compared with non-kinked catheters [7]. Furthermore, the small experimental sample size and number of EVD insertions decreases the statistical power of our study and decisive conclusions cannot be reached. Finally, for definite validation of its viability, testing of the method versus other and well-established techniques is of utmost importance.

## Conclusions

The idea of useful medical apps uploaded on conventional mobile phones as portable and convenient operating tools for neurosurgeons led us to evaluate a smartphone device as a clinical guiding instrument for EVD insertions. Our data suggests that smartphone-assisted adjustment of the lateral insertion angle allows reliable EVD placement tailored to the individual patient.

## Abbreviations

app: Augmented-realty mobile device application
cm: Centimeter
CT: Computed tomography
DICOM: Digital imaging and communications in medicine
EVD: External ventricular drain
Fig: Figure
M: Mean values
PACS: Picture archiving and communicating system
SD: Standard deviation
sEVD: Smartphone-navigated placed external ventricular drain
